# Spider Bite

**Published:** 1860-09

**Authors:** J. T. Banks

**Affiliations:** Griffin, Ga.


### ATLANTA

### Medical and Surgical Journal.

VOL. VI.] SEPTEMBER, 1860. [NO. 1.

### ORIGINAL COMMUNICATIONS.

### ARTICLE I.

*Spider Bite.—Severe Symptoms and Unusual Phenomena—Recovery.* By J. T. BANKS, M. D., Griffin, Ga.

On the 8th of July I was summoned to Mr. F.'s, to see his little son (Lewis), between five and six years old. Lewis had been bitten by a spider on his buttock, near his anus, about forty or fifty minutes before my arrival. The place bitten presented the usual appearance of places stung or bitten by poisonous insects—a small, white, elevated spot, circular, and about the size of a five-cent piece. There was no surrounding swelling or redness; the little whale soon disappeared. His mother had bathed the location in Darbey's Prophylactic Fluid, (a chlorinated mixture.) The pain in the bitten part lasted about ten minutes; after which, he cried of pain in his stomach, of an intermitting character; the intermissions were from thirty seconds to one minute long; and the paroxysms of pain were from ten to fifteen minutes long. His abdominal muscles were rigidly contracted—face and neck very red, the vessels of which were much distended; circulation slow and feeble, as under the influence of a powerful sedative; perspiration free; nausea, with frequent efforts to vomit—seeing which, his father gave him a dose of an emetic mixture that he kept in his family for croup, from his notion that, if he could vomit freely he would be relieved of the nausea.

After satisfying myself of the nature of the case, I gave him of good whisky, ℥ i.; laudanum, 6 to 8 gtts., and ordered warm fomentations to his abdomen; and as soon as I could obtain it, commenced and gave him Aqua Ammonia and laudanum combined, every half hour, and as often about ℥ i. of whisky: this treatment was continued four or five hours, without any appreciable benefit, the little sufferer having taken from 60 to 80 drops of laudanum. I then determined to freely open his bowels, continuing the whisky and ammonia without the laudanum.

After he had been bitten two hours, I was examining his abdomen, to see if the intermissions of pain were attended by a relaxation of the abdominal muscles, and after satisfying myself that there was no appreciable relaxation, by accidental touch my attention was drawn to his penis, which I found in *perfect erection.* Soon after I discovered the erection of his penis, he began and made frequent ineffectual efforts to micturate. Without any effort to relieve him, and much to the discomfort of his parents, I permitted this very distressing condition to continue about an hour, until I became satisfied that his failure to empty his bladder depended upon the continuous perfect erection of his penis. I then directed my efforts to its reduction, which was accomplished in ten or fifteen minutes by wrapping his penis in tobacco, previously softened in warm water, extending the application of the tobacco to his perineum. As soon as his penis limbered down under the influence of the tobacco, he had a free and copious flow of urine. After the effects of the tobacco had subsided, the priapism returned and continued, with only a few remissions, so long as his system was under the influence of the poison.

The free evacuation of his bowels gave no relief to his pain. After he had suffered eighteen or twenty hours, the irritation of his rectum and bladder became so distressing, causing so many calls to the bed-glass, I was compelled to give him cold laudanum injections for their relief.

During his second day's illness, the intermissions from pain increased in length, the swelling of his eyelids and the redness of his face and neck began to improve, and his circulation to respond to the use of stimulii. Stimulants were given often, but only as his circulation and pain demanded, for whisky, in ℥ i. doses, gave him more relief than anything else, and only after taking it could he get a few minutes sleep.

The severe pain in his stomach was soon attended with much tenderness on pressure, but subsided with the final subsidence of the pain.

In his third day the intermissions from pain were perfect and of longer duration; so were the paroxysms of pain longer, but without the corresponding diminution of severity, and often located in his teeth instead of his stomach. His circulation was increased, and he had an inordinate hunger; at the close of nearly every paroxysm, he would cry out for something to eat, and as it produced no increase of pain, he was allowed a liberal quantity of coffee, light cake, butter, etc. His desire for food seemed to be increased by the poison, for he would often cry out for something to eat during his first and second day's suffering—more than the amount requisite to supply the natural want. I then determined to try the Ext. Belladonna and Quinia—the first for its anodyne effect, and the latter for its neurotic—in combination with Ipecac, to meet the increased excitement. I gave of Ext. Belladonna gr. $\frac{1}{8}$, Quinia Sulp. grs. 5, Ipecac gr. i., every two hours, until he had taken three doses, but without any effect. In the fourth dose I lessened the amount of Quinia, and increased the Ipecac to 5 and 8 grs., which produced emesis in about one hour; after which he had a quiet sleep of four hours. His convalescence was rapid from that time, only taking two or three more doses of his prescription, at longer intervals, and continuing the whisky from three to five times per day, for a few days.

No spider could be found in the privy, where he was when bitten, but he says he saw the spider; that it was a large, black spider, that had webbed itself across the seat of the privy, where he sat down to have an operation. His father has since informed me that he has seen it several times since in the privy, and at one time appeared disposed to make fight. He describes it as a large, black spider, with a vicious appearance; the body about the size of the end of his little finger, round and plump; and when its legs are extended it would cover an area between the size of a quarter and half dollar piece. Lewis was convalescing from Measles followed by Relapse fever, from which he had been able to walk about but a short time. His absorbents were in *hungry action*, which facilitated the very rapid diffusion of the poison throughout his system. In no case that I can find reported has the poison diffused itself throughout the entire system in so short a time.

The case reported by Isaac Hulse, M. D., U. S. N., in the twenty-fourth Vol. American Journal of Medical Science, May No., 1839, the poison was an hour diffusing itself, as evidenced by the symptoms. In the case reported by Dr. J. C. Cross, of Courtland, Ala., in the Transylvania Journal of Medicine, and republished in the New Orleans Medical & Surgical Journal, Vol. 16, 1859, November No., the poison manifested signs of diffusion during the second hour. In no case has the bite been followed by local inflammation. The unity of the poison is clearly established by the commonness of effect. They all tolerated stimulents and narcotics alike; pain of an intermitting character; greatest intensity in the abdomen; and all attended by irregular muscular action.—Dr. Hulse gave his patient four ounces of laudanum and the same of aqua-ammonia in four hours. Dr. Cross gave two drachms of laudanum in an hour and a half after trying the effect of opium otherwise. Another remarkable coincidence in these cases is, that they were all bitten in the temple of Cloacina; two of them on the same part. The imperfect discription of the spider, given in these cases, together with their abode, makes them members of the same specie of Arachnida. Although we have but few cases reported of severe and threatning symptoms, from the bite of any specie of Arachnida common in this region of country, they are of sufficient importance to claim more of our professional attention, that the public may be better informed of their danger. I have regarded the mode by which this species of Arachnida inflict the wound into which the poison is deposited as a bite, the correction of which I can not substantiate from my limited knowledge of the natural history and anatomical construction of the species. Some of the species of this family are said to sting, (the Scorpion) others are differently characterized as biting by some, and stinging by others; (Tarantula), others say that they inflict the wound by piercing the skin with its forceps, and at the same time eject from its mouth the poison in the wound, (Rus's Cyc) and still others say that each tooth or piercer is perforated by a tube leading to a sack of poison, through which the poison is passed. Will not some student of nature investigate the habits and anatomical construction of this species of spider, and give us the benefit of their labor?

